# Gleanings from Our Exchanges

**Published:** 1873-01-01

**Authors:** 


### Gleanings from Our Exchanges.

### PRURIGO AND PRURITUS.

Dr. Rothmund (*Aert. Intell. Bl.*, 26th Sept.) says that prurigo and pruritus are old-fashioned names for two essentially different skin diseases, which have been most incorrectly confounded with each other very frequently. They are certainly etymologically in so far alike that they both mean itching, and itching is common to both of them. The essential difference consists in this, that prurigo manifests itself by the eruption of abundant intensely itching knots, whereas in pruritus no naked-eye alterations of the skin are visible. Both are more frequently diagnosticated by second-rate physicians than they actually are seen in nature; and they are occasionally both confounded with lichen.

The knots of prurigo are generally noticed on the extensor sides of the lower and upper extremities, but seldomer and less observably on the trunk. Well-marked forms of prurigo are rare in Germany. As these knots of prurigo call for scratching, from the intense itching they cause, we find usually their summits wounded and covered with a crust, from the issue of a drop of blood. For the same reason we almost constantly meet with an artificial eczema, and also a marked swelling of the inguinal and crural glands, which is partly caused by the eczema. As this form of disease is caused by a chronic hyperæmia and inflammation of the papillæ of the skin, we have to enroll it among the inflammatory processes.

On the other hand, we must consider *pruritus* as an anomaly of the nerves of the skin. Here the skin exhibits no marked efflorescence, neither vesicles nor knots, nor other phenomena, which can be ascribed to the scratching — erythema, excoriation, or eczema. We generally find this affection unaccompanied by any notable affection of the health, but occasionally we find it accompanying Bright's disease or jaundice in rare cases. The cause of this rare but peculiar fact lies in obscurity; retention of urea or coloring matter of the bile cannot be laid down as the cause.

Sometimes the abnormal itching, which robs the patient of sleep and appetite, is extended over the whole body.

The seat of the affection is sometimes rather variable. Of pruritus, at least, we cannot say that it is found more on the hard parts than on the mucous surfaces; but it often limits itself in the most troublesome manner to the organs of generation. Prurigo is not uncommon in childhood; but pruritus, on the contrary, is proportionally far more common in old age.

The treatment of these two affections was for a long time a true crux medicorum, and the number of therapeutic agents which have been employed, and the constant attempt to suggest new remedies, shows this sufficiently. Had any of the remedies recommended been of great utility, all the rest would have been thrown aside. Here, as in many other diseases, the greater the number of remedies, the greater the certainty that none possesses truly the boasted power, and none is able to maintain the trust we bestow on it.

I will not go further into the history of the therapeutics of these two affections. I count merely on the fingers those hitherto used: Cold effusions, sea-bathing, salt baths, sulphur soap, sulphur and sand soap, sulphur ointment, sublimate baths, alcoholic lotions, veratrus (very dear), chloroform, tar ointment, arsenic, etc. To all of them we can, more or less, only ascribe the term of palliative; and yet our ancestors were certainly not bad therapeutists—perhaps much better than our successors will be. Of all the above-named remedies, long-continued, energetic chloroform or tar ointments give satisfactory results in mild cases. Ether preparations give some hours' peace; and rubbing with brandy in like manner is useful. For a certain cure, however, the difficulty lies in this —how to prevent relapses, which come on sooner or later, or how to arrive at lasting results. The most suitable for this was the long-continued use of tar preparations (in form of tar soap, sulphur-tar soap, oil of tar, or spirit of tar, or pix liquida). This process, however, had much against it, for it is unsuitable in thick, dry, or tender skin, and dirties the linen terribly.

The not unfavorite internal use of arsenic (as Fowler's solution, or Wilson's liq. ferri. arsen.) I have (although I have learnt to prize it greatly in non-syphilitic psoriasis) found always an unlucky weight on the organism. In childhood, the use of iodized cod-oil deserves all praise, and is rarely contraindicated.

In No. 41 of last year's Journal (pp. 502–505), I spoke shortly concerning the treatment of a disease which was quite obscure until this century. At that time I also spoke of the favorable influence of carbolic acid upon prurigo, and especially on pruritus, adding that this acid worked from the blood in the peripheral nerves. This observation I do not retract now, but I have to add one more proved and tried.

The internal administration of carbolic acid excels in good effects in this affection that of any other body in the Pharmacopœia. It is an effective cure, and not merely a palliative. At first I gave it in the form of pills, six centigrammes in each, one twice a day; but the disagreeable smell of the acid was against it. This was lessened by using carbolate of soda, which is a mild and useful preparation.

When I had gradually found that this preparation was tolerated in rather high doses, and that it did not contradict the desired effect I hoped for, I felt myself compelled to try how it would act in the form of subcutaneous injections.

The last-named experience appears to me, after further observation, to present such advantages that I publicly announce that it will take fast root in the therapeutics of skin diseases, and that it will not be unworthy of the greatest attention.

The accounts published by Neumann, of Vienna, as well as the consequences drawn from them, made me observe great care at the commencement of my experiments. But after long trials, all fear quickly vanished, and even the early fear I had that injections might call forth great local irritation, I have given up as erroneous. In a great number of injections made by me, I have never noticed any pain out of the common, nor any peculiar affection of the skin. At first, at each injection, a newly-gilt needle was used; but later, cleansing of the needle used was found quite sufficient. Solutions of pure carbolic acid appeared more efficacious than those of carbolate of soda. The solution contained 25 centigrammes of carbolic acid and 30 grammes (four grains to the ounce) of water, so that each time about one centigramme of carbolic acid was injected, or less.

We must not omit to remark that the hypodermic method is so much the more to be preferred to the internal use of the remedy, because it causes a more rapid effect, a much less quantity of the substance is needed, and the digestive organs are left intact, thus sparing the nutrition of the patient.

According to what I have said, I hold myself entitled in every respect to describe the favorable effect of carbolic acid in prurigo, and especially in pruritus, as no longer doubtful, and to recommend further trials of hypodermic injection, in such cases, with carbolic acid.—*The Doctor.*

---

Dr. R. W. Gibbes, of Columbia, has been elected Professor of Anatomy and Surgery in the University of South Carolina, in place of Dr. Darby, resigned.

### CLINICAL EVIDENCE IN FAVOR OF THE VALVULAR ORIGIN OF THE HEART'S FIRST SOUND.

The production of the first sound of the heart is now very generally referred to the vibratory tension of the mitral and tricuspid valves. Some authors, however, still contend for the partial production *at least* of the sound by the muscular bruit and the movement of the blood. Prof. Bamberger, who is one of the leading German authorities on diseases of the heart, some little time back published (*Wien. Med. Wochenschr*, No. 1, 1872) a very interesting account of two rare affections of the heart which bear closely on the mechanism of the production of the first sound. An abstract of this paper appeared in the *Centralblatt* (No. 16, p. 2,147 *et seq.*), which we translate for our readers.

Bamberger is of opinion that the chief objection against the experiment of Ludwig and Dogiel is by no means refuted—the objection, namely, that a valvular sound may arise in a heart which is devoid of blood. He thinks *prima facie* the first sound must be valvular because of the great similarity between it and the second, of which the valvular origin is not disputed. Again, the assumption that it is a combination of a muscular and valvular sound is not justified, for the first sound is clear, distinct, and sharply circumscribed. He, however, concedes the existence of a muscular sound in cases of hypertrophy and of excessively increased normal action; but this muscular sound is essentially different from the normal sound and not difficult to distinguish. Von Bamberger mentions that he had under care in the hospital two cases of great hypertrophy (one accompanying mitral stenosis, the other aortic insufficiency), in both of which the muscular sound could be heard without the stethoscope at a distance of two feet, a thing which never happens with valvular sounds.

Two other very interesting cases have furnished new proof in favor of the view that the first sound of the heart is a valvular sound.

The first of these was that of a day laborer, aged 40, the subject of chronic catarrh, emphysema, and dilatation of the right heart. Dropsy and the usual symptoms of chest embarrassment had supervened. Two circumstances only made the diagnosis at all doubtful. The ailment had existed twenty-three years, and had begun with ascites. Sectio cadav. disclosed bronchitis and dilatation of the heart; the former, however, was insignificant, and the latter had arisen from other causes. The chief feature of the case was a very peculiar form of malady which the author has already seen in other instances, but which has not been hitherto described. He cannot as yet draw a precise pathological picture of the affection, but it consists in chronic inflammation that attacks almost all the serous membranes in succession, occurring without any recognizable special cause, or any previous constitutional dyscrasy, and pursuing its course with exceeding slowness. It is accompanied by considerable thickening and indurative degeneration, but there are no local inflammatory or irritative symptoms, or at any rate none of importance. Frequently, but not always and not in all situations, it gives rise to considerable serous effusions.

In the case above-mentioned the author found extensive induration of the peritoneum, of the capsules of the liver and spleen, of the pleura, and especially of the pericardium. The latter membrane was free on the lower part only of the anterior surface of the left ventricle: everywhere else it was adherent, and was changed into an immoveable capsule of stony hardness, from which processes projected into the muscular tissue of the heart. Thin sections under the microscope show it to be merely calcified connective tissue. On being treated with hydrochloric acid the stroma of the connective tissue remains. The capsule is made up of two halves, which are articulated over the right ventricle like the two halves of a mussel-shell. Both auricles and the venous orifices were extraordinarily wide, the whole heart considerably enlarged, the valves unaltered. Heart tissue brownish in color, being in the condition of brown atrophy ("simple" atrophy?); the thickness of the muscular wall at the site of the non-adherent surface of the left ventricle amounted to nine millimeters (=0.354 inch).

In this case the first sound was always distinct and quite clear over the apex and tricuspid space. Von Bamberger thinks it impossible to suppose that the muscle which, with the exception of a part of the front of the left ventricle, was entirely incorporated with a rigid calcareous shell that gave off numerous projections into its substance, and which was itself in a condition of brown atrophy, could have produced a distinct and clear sound. On the other hand, there was no hindrance here to the production of the sound by the valves.

The second case was that of a day laborer 21 years of age, who up to a year previously had always been healthy. Since then, however, every time he did any work that required more effort than usual he suffered from violent palpitation and severe oppression of the chest. This oppression quickly increased in severity, came on spontaneously, and finally compelled him to go into the hospital. Here the following symptoms were noted: slight cyanosis of lips; no dropsy; area of heart's action enlarged, as evident both to sight and touch; apex impulse in the fifth intercostal space, three centimetres (=1.18 inch) outside the nipple line, and considerably increased in force; absolute dulness from the site of the apex impulse to the upper edge of the third left cartilage, and from the middle of the sternum to nearly two fingers' breadth beyond the nipple line; at the apex a very loud murmur is heard of peculiar quality (*timbre*) occupying the whole of the systolic period, whilst during diastole a very short, weak murmur is heard in the same situation; over the tricuspid area the murmur is heard, but less audibly; second sound normal; aortic sounds normal; second sound of pulmonary artery distinctly exaggerated; pulse wave low, arterial tension slight. The diagnosis was, mitral insufficiency and stenosis of the venous orifices—given with hesitation. The patient quickly sank under an attack of pneumonia.

The heart was much hypertrophied, especially in the transverse measurement. Left side very large; walls of left ventricle soft; wall of right ventricle firm, but not so thick; the wall of the right conus arteriosus four millimetres (= 0.157 inch) thick, with great fatty degeneration; no fatty degeneration anywhere else; musculi papilares of right ventricle decidedly hypertrophied — of left ventricle slightly so; valves all normal, except the edges of the extremities of the mitral, which are thickened a little, and some slight knotty enlargements on some of the chordæ tendinæ. In the left auricle, and projecting into the auriculo-ventricular orifice, there is a club-shaped mass, forty-two millimetres (= 1.65 inch) long, forty-one millimetres (= 1.61 inch) broad, and twenty millimetres (= 0.787 inch) thick, which is connected by a thin pedicle, at a point about thirteen millimetres (= 0.511 inch) from the base of the posterior flap of the valve, on the line between the pulmonary veins of the two sides. The mass is glassy and jelly-like, and under the microscope resembles myxoma.

Here was a case in which during life a prolonged systolic murmur was heard, with no trace of the normal sound. After death, no alteration of the muscular structure of the heart worthy of note was to be discovered. On the other hand, a tumor was lying upon the auricular surface of the mitral valve. The fact that this was sufficient to produce a systolic murmur shows that the systolic sound must be a valvular sound, for if it were a muscular sound the tumor could not have had any influence upon the formation of the sound. But the natural sound was interfered with because the regular sound—producing oscillatory movement of the valves—was interrupted by contact with the tumor.—*The Doctor.*

Treatment of Diphtheria by Inhalations of Sulphur.—Dr. Kieser recommends, in the *Wurtemburg. Medicin. Correspond.-Blatt*, the treatment of diphtheria introduced originally by M. Barbosa, viz., insufflations three times a day of the commercial flowers of sulphur, by means of a little cone of paper, the bent end of which is charged with the drug. Ordinarily three to six insufflations are sufficient, in cases seen early, to cause the disappearance of the exudation, and the cure is complete in from eight to ten days. In neglected cases nine to twelve insufflations are required, and the cure requires twelve to fourteen days. Dr. Kieser thinks that nurses can be readily taught to practice the insufflations, and in young infants he advises a glass tube with one end fine and bent, and the other armed with an india rubber bulb, to be substituted for the cone of paper. We cannot think insufflations should be left to nurses, and with infants the danger of glass tubes is manifest.—*The Doctor.*

Varicose Veins.—Dr. S. Soboroff described in *Virchow's Archiv.*, liv. 1-2, p. 137, the structure of varicose veins. The chief change he observed was hypertrophy of the smooth muscle fiber. There was no change in the epithelial coat. This layer was perfectly intact, though several cells seemed enlarged in dimensions. It is probable that this process is connected with a hyperplasia of the cell elements. The other peculiarities were not so constant. In some cases there was increase of the muscular tissue; in others, a fuller development of the vasa-vasorum.—*The Doctor.*

